# Chronic mechanical trauma/irritation and oral carcinoma: A systematic review showing low evidence to support an association

**DOI:** 10.1111/odi.14049

**Published:** 2021-10-20

**Authors:** Monica Pentenero, Lorenzo Azzi, Giovanni Lodi, Maddalena Manfredi, Elena Varoni

**Affiliations:** ^1^ Department of Oncology Oral Medicine and Oral Oncology Unit University of Turin Turin Italy; ^2^ Department of Medicine and Surgery Unit of Oral Medicine and Pathology University of Insubria Varese Italy; ^3^ Department of Biomedical, Surgical and Dental Sciences Università degli Studi di Milano Milan Italy; ^4^ Department of Medicine and Surgery Dentistry Unit University of Parma Parma Italy

**Keywords:** chronic mechanical trauma, mucosal irritation, oral squamous cell carcinoma, risk factors

## Abstract

**Background:**

Chronic trauma of oral mucosa, resulting from repeated and persistent mechanical irritative action of an intraoral injury agent, has repeatedly been reported to be possibly implicated in the development of oral squamous cell carcinoma (OSCC).

**Objectives:**

The present systematic review aimed to assess whether chronic mechanical trauma can be considered a risk factor for OSCC.

**Data sources:**

PubMed, CENTRAL (Cochrane Central Register of Controlled Trials), Scopus; EMBASE, Web of Science.

**Study eligibility criteria:**

Cohort studies comparing OSCC incidence among subjects with/without chronic mechanical trauma or case–control or cross‐sectional studies comparing chronic mechanical trauma among subjects with/without OSCC.

**Results:**

Only one prospective case–control study fulfilled the inclusion criteria, but the quality of the evidence provided is not enough to define trauma as a risk factor for OSCC. The main limitation is the presence of only one case–control study at high risk of bias.

In the absence of strong evidence supporting the role of trauma in OSCC, a thorough discussion on trauma and carcinogenesis has been performed.

**Conclusions:**

Available evidence does not support an active role for chronic trauma in oral carcinogenesis, neither as promoter nor as progressor factor. Prospective cohort studies able to better assess trauma in OSCC are needed.

## 1 INTRODUCTION

Oral squamous cell carcinoma (OSCC) is the most frequent malignant neoplasia of the oral cavity.

Several risk factors have been associated with the development of OSCC; first of all, smoked or chewed tobacco (with or without betel/areca nut) as well as alcohol consumption are currently considered the two most important risk factors implicated in the aetiology of OSCC (Jemal et al., 2011).

High risk oncogenic human papillomaviruses (HPV) are also associated with oropharyngeal squamous cell carcinoma, with increasing incidence in developed countries (Gillison et al., 2015).

In addition to these well‐recognized risk factors, patients’ immunosuppression, malnutrition or dietary deficiency, poor oral hygiene and predisposing genetic factors have been proposed to be significantly involved in the genesis of OSCC (Shaikh et al., 2015).

Chronic trauma of oral mucosa, resulting from repeated and persistent mechanical irritative action of an intraoral injury agent, alone or in association with other risk factors, has also been reported to be possibly implicated in the development of OSCC (Lockhart et al., 1998; Piemonte et al., 2010,2018; Thumfart et al., 1978; Warnakulasuriya, 2009).

However, the exact role of this potential risk factor has not been clearly elucidated, yet. The aim of this review is to evaluate the current literature in order to assess whether chronic mechanical trauma can be considered a risk factor for OSCC.

## 2 METHODS

This study followed the guidelines of the PRISMA statement (Liberati et al., 2009). The review protocol was registered in PROSPERO under the number CRD42018100178.

### 2.1 Question

Is chronic mechanical trauma a risk factor for squamous cell carcinoma of the mouth?

### 2.2 Electronic search

A systematic literature search was conducted with the following databases: PubMed, CENTRAL (Cochrane Central Register of Controlled Trials), Scopus, EMBASE and Web of Science. We did not place any restrictions on the language or date of publication when searching such databases. The last search was performed on January 3, 2021.

A query was designed to search PUBMED and then adapted for the other databases as reported in the Supplementary appendix. Also, reference lists of previous meta‐analyses and other relevant papers were searched. All abstracts were reviewed independently by at least two of the authors to determine whether studies met the inclusion criteria. We resolved any disagreements by discussion. When the article was considered relevant by the two reviewers, the full papers were obtained and evaluated.

### 2.3 Inclusion criteria

#### 2.3.1 Study design

Cohort studies comparing OSCC incidence among subjects with and without chronic mechanical trauma or case–control or cross‐sectional studies comparing chronic mechanical trauma among subjects with and without OSCC.

#### 2.3.2 Participants

For cohort studies: healthy subjects exposed to chronic mechanical trauma.

For case–control and cross‐sectional studies subjects with a definitive (histological) diagnosis of OSSC.

#### 2.3.3 Controls

For cohort studies: healthy subjects not exposed to chronic mechanical trauma.

For case–control and cross‐sectional studies: subjects without oral cancer.

#### 2.3.4 Exposure

Chronic mechanical trauma, as demonstrated by the presence of a traumatic factor either dental (tooth malposition, diastema, sharp or broken tooth, sharp or rough fillings or fixed prosthesis), prosthetic (sharp or rough dentures, defective retainers, overextended flanges, denture without stability and/or retention) or functional (tongue interposition, lip/cheek/tongue biting or suction, dentures stabilization with tongue, etc.). The patient's recollection of an history of chronic trauma was not considered reliable enough in order to define the presence of exposure.

### 2.4 Data extraction

A standardized data extraction form was prepared and tested for review of three articles independently by three reviewers. Eligibility, validity and design information will be recorded on the extraction form for each study by two of the authors. In case of discrepancies, we resolved any disagreements by discussion.

### 2.5 Risk of bias

The same two authors who extracted the data assessed the risk of bias of the included studies employing the proper JBI Critical Appraisal Checklist depending on the study design (Joanna Briggs Institute, 2021).

### 2.6 Data synthesis

The association between OSCC and chronic mechanical trauma was estimated by calculating OR and the 95% confidence interval (CI). When absence of events in one of the groups caused problems with computation of OR, 0.5 was added to all values for that study, except when absence of events involved both study and control groups, in this case OR was undefined (Yu et al., 2010). Since heterogeneity among studies was expected, a random effect was used to calculate the summary estimate using the Mantel–Haenszel method (Greenland, 1985; Mantel & Haenszel, 1959).

A sensitivity analysis was planned, excluding studies of lower methodological quality (i.e. studies at high risk of bias).

## 3 RESULTS

### 3.1 Literature search

**FIGURE 1:**
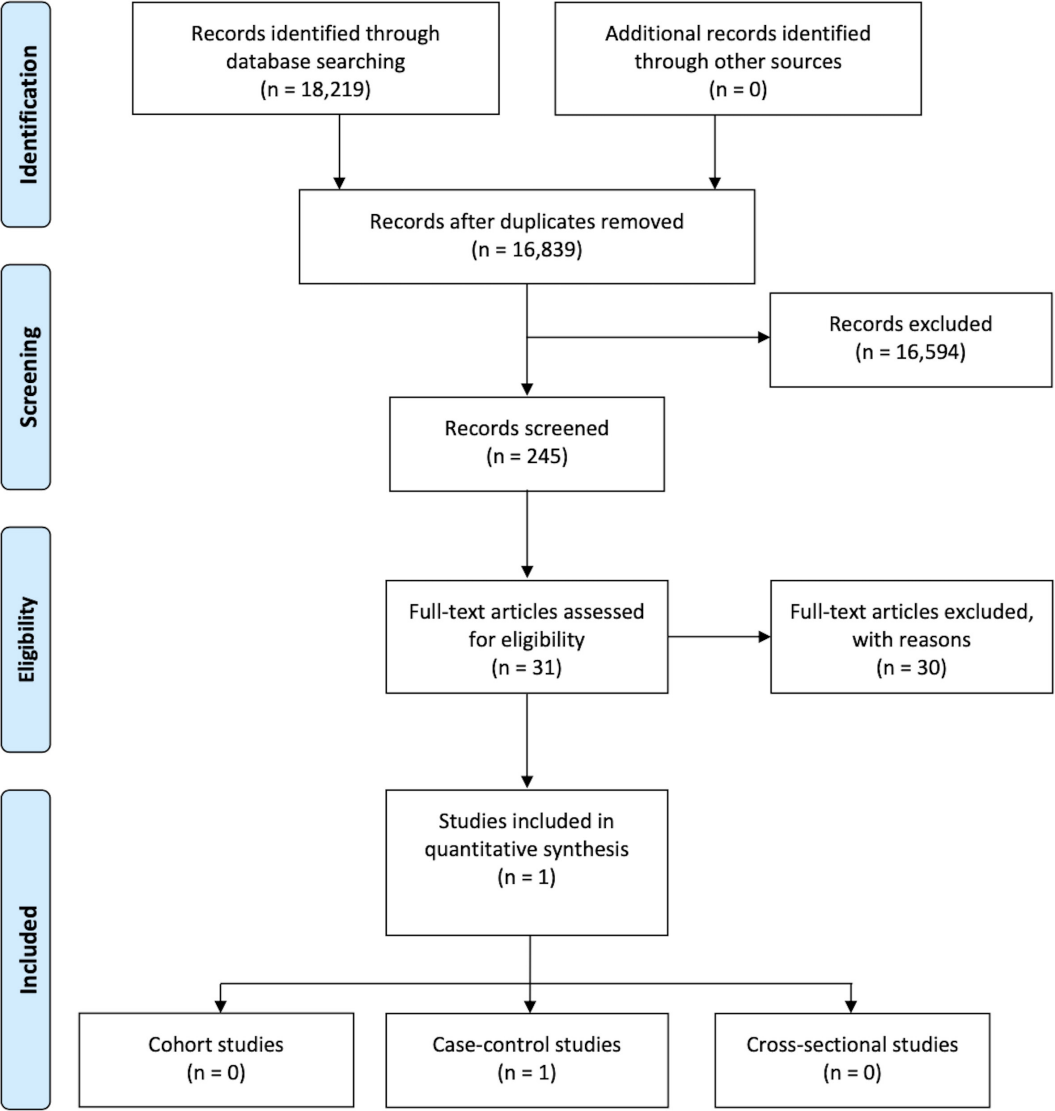
PRISMA flow diagram

The study selection process is summarized in Figure 1. The search strategy in the databases resulted in 18,219 records. Last search was conducted on January 03, 2021. A total of 1380 records were cited in more than one source (duplicates). The authors independently screened the title and abstract of the papers related to the subject being studied: 16,808 records were excluded for not being related to the topic. The eligibility of the resulting 31 articles was assessed by full‐text analysis, and 30 papers were excluded because they did not meet the inclusion criteria (Supplementary appendix). Finally, only 1 paper was included in this review.

### 3.2 Risk of bias of the included study

The included study (Piemonte et al., 2018) was judged at high risk of bias (Supplementary appendix). Cases and controls were not comparable other than the presence of the disease. The authors state that controls were patients with similar age range, but significant differences between the groups were observed for age and gender. The assessment of the exposure was not considered valid and reliable, and the exposure was not measured in the same way for cases and controls as discussed below. It was not possible to establish whether a consecutive recruitment was conducted.

### 3.3 Presence of trauma

The included study (Piemonte et al., 2018) reported that 72% of OSCC patients (i.e. 38/53) developed lesions associated with trauma, while only 36% of the patients included in the control group (i.e. 36/100) had lesions associated with trauma (OR = 4.5; CI:2.2–9.2; *p* = 0.0001). Of note, the numbers of exposed cases and controls are not consistently reported thought the paper as described in the Supplementary appendix.

There were neither gender predilection (OR = 0.45 CI: 0.18–1.15; *p* = 0.099), nor differences in the subgroups identified in the sensitivity analysis for the other risk factors (never smokers OR = 4.5; CI:1.3–15.2; *p* = 0.013; smokers OR = 4.1; CI:1.6–10.5; *p* = 0.0016; never drinkers OR = 7.07; CI:1.78–28; *p* = 0.002; drinkers OR = 4.04; CI:1.6–9.9; *p* = 0.002).

Interestingly, there were no differences in trauma occurrence in relation to tumour size (OR = 0.88; CI:0.26–2.94: *p* = 0.84).

### 3.4 Time of exposure

The included study reported data about the time of exposure to trauma (Piemonte et al., 2018). The overall data were not reported as the time of exposure was only reported subdivided for males and females. In both genders, the oral cancer group reported significantly longer time of exposure to chronic mechanical irritation (M–OSCC group average 62.4 months, Std Error 95.4, vs control group average 25.8 months, Std Error 54.1; *p* = 0.0047; F–OSCC group average 114.5 months, Std Error 165.2, vs control group average 34.7 months, Std Error 80.4; *p* = 0.0013).

## 4 DISCUSSION

In the last 10 years, an association between the onset of OSCC and the presence of chronic trauma of the oral mucosa defined as repeated mechanical irritative action due to several conditions has been hypothesized. Particularly, chronic trauma could be suspected in the presence of teeth characterized by sharp or rough surfaces, ill‐fitting dentures or parafunctional habits. Quite frequently the presence of trauma had been reported just on the basis of the patients’ referral, leading to unreliable data which were excluded in the present review.

Most of concern about the literature on chronic trauma and OSCC just arises from the same definition of trauma leading in our opinion to an important overestimation of its association with OSCC. Many studies were excluded for the lack of any clinical information about CMI (Bektas‐Kayhan et al., 2014; Garrote et al., 2001; Guha et al., 2007; Marshall et al., 1992; Rosenquist et al., 2005; Talamini et al., 2000; Zheng et al., 1990,1992). For this reason, we applied in the present review highly restrictive criteria to define exposure to chronic trauma aiming to collect the most reliable evidence. Piemonte and Lazos, often co‐workers, report the most complete definition available in the current literature (Lazos et al., 2017,2019; Piemonte et al., 2010,2018). Nevertheless, it may imply an overestimation of exposure.

The presence of chronic mechanical irritation (CMI) is detected in any case of erythema, atrophy, ulceration, keratosis, hyperplasia, indentation or fibrosis in direct contact with a ‘mechanical agent’ (e.g. teeth or denture), during functional/parafunctional movements or decubitus position. This means that most if not any mucosal lesion of the border of the tongue can be associated with CMI: in rest position, the whole border of tongue is in direct contact with teeth or denture if present. Similar considerations apply to large part of the vestibular mucosa. Most of the oral mucosal surfaces have physiological contacts with teeth or dentures during functional movements or in rest position, so that the required direct contact between the traumatic agent and the lesion can be easily observed and even more in the presence of OSCC often implying altered mucosal surfaces due to an increased volume. In the presence of OSCC, a trivial contact with teeth or denture represents a CMI, but when assessing control patients, the same light contact is considered a CMI only in the presence of an objective clinical lesion. As any kind of light contact (due to interposition in the presence of diastema, or to lingual inclination of teeth, or to the habit of stabilizing the denture using the tongue) implies very rare (if any) clinical lesions, this results in overestimation of CMI in OSCC patient and underestimation of CMI in control subjects. Additionally, all sources of trauma were jointly included, without considering the different impact of dental/prosthetic trauma when compared to parafunctional habits. More recently, in order to achieve rigorous methodological standards, the same authors stated that ‘a tooth/denture could be a chronic mechanical irritation agent if and only if it actually produces a lesion on the oral mucosa’ (Lazos et al., 2019). It means that in the absence of lesion no CMI can be observed: this creates a short circuit preventing to know which came first, lesion or trauma? To solve this issue, the same authors assessed the presence of the potential traumatic agent before the onset and/or modification of the lesions through anamnesis. This leads to an important recall bias due to both rethink with greater intensity than controls on all the possible events that may have caused the disease and to the renown defective self‐perception of OSCC patients frequently leading to delay in perception of lesions. An important recall bias can also arise from studies which defined the presence of trauma just from questionnaire or patients’ referral (Guneri et al., 2005; Jain et al., 2016; Rotundo et al., 2013; Vaccarezza et al., 2010; Velly et al., 1998; Winn et al., 1991; Yan et al., 2017). For such unavoidable criticisms, the criteria adopted in the present review to define the exposure did not take into account patients’ recollections or patients‐reported chronic trauma. Irrespective of the definition of chronic trauma, if a carcinogenic effect has to be suspected, it must be assumed that trauma lasts for a long time as neglected by patients. This could likely occur just in the absence of symptoms which nevertheless are invariably observed in the presence of trauma induced by sharp teeth or ill‐fitting denture. In the absence of reported symptoms, the presence of a contact rather than a significant trauma should be assumed. Therefore, lacking a proven constant presence of trauma before the onset of OSCC, it is unlikely that the detection of sharp teeth or ill‐fitting denture topographically related to OSCC at the time of diagnosis could have had any role in carcinogenesis. Other authors questionably reported the presence of trauma in normal individuals, even in the absence of very sharp teeth, due to the presence of dental cusps which are by nature pointed structures. Such a large contact area between tongue and teeth would make sense of field cancerization (Panta et al., 2018). All these clinical considerations could make difficult, if not impossible, a prospective observational study aimed to assess the role of CMI in oral carcinogenesis. May be some data could be acquired from the observation of clinical conditions as frictional keratosis, linea alba or morsicatio buccarum, but currently all of them are recognized as non‐part of the oral potentially malignant disorders (Warnakulasuriya et al., 2020).

**FIGURE 2:**
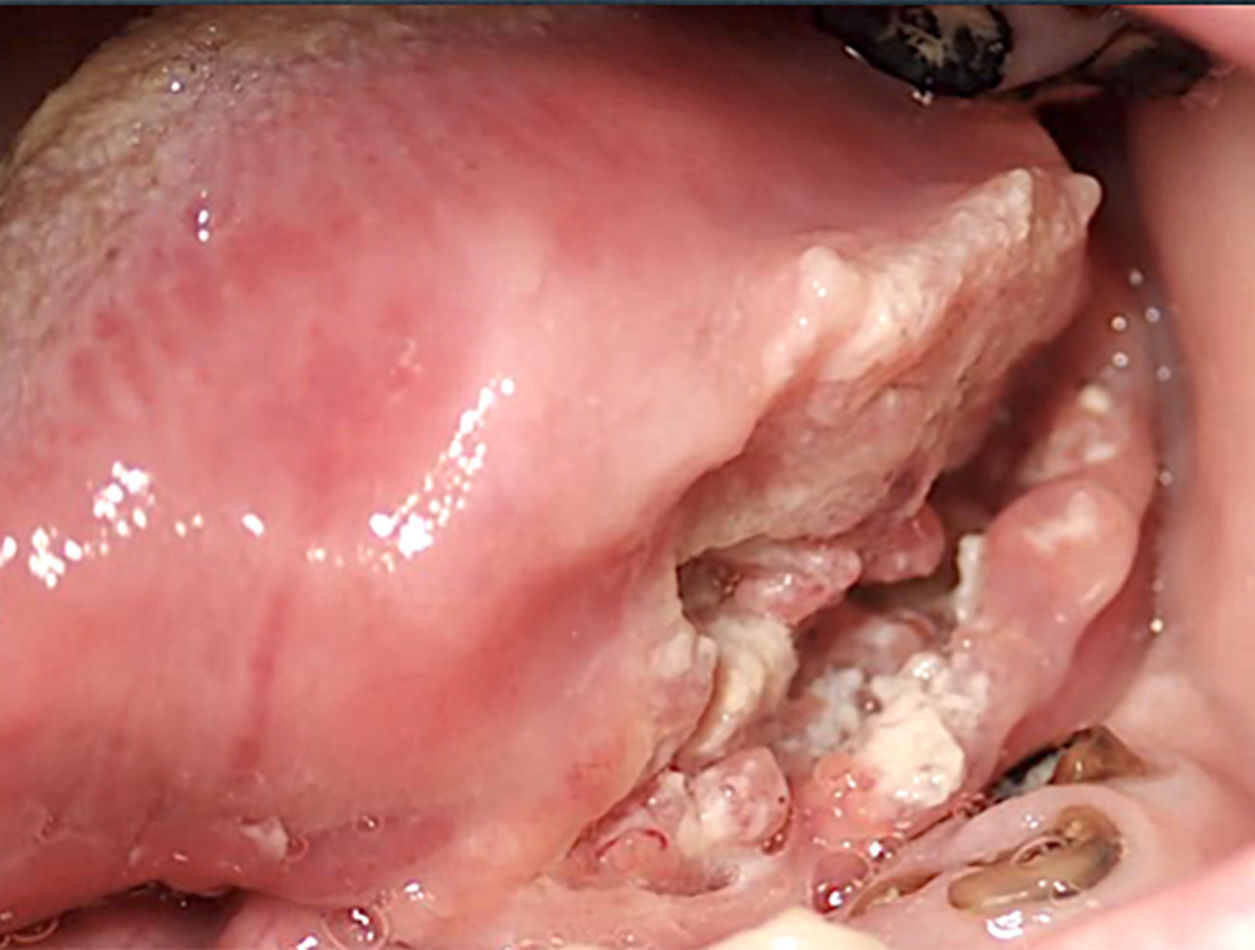
Oral squamous cell carcinoma involving the lateral tongue and the floor of the mouth and topographically related to the presence of residual tooth roots

The scientific interest on the potential association between CMI and OSCC started from the trivial observation of neoplasms occurring in areas in contact with teeth and/or dental appliances (Figure 2) which lead some authors to support the role of chronic dental trauma in the causation of mouth cancer, even in the absence of any difference in dental and prosthetic factors when comparing patients with intraoral versus extra‐oral head and neck tumours (Lockhart et al., 1998; Thumfart et al., 1978). Very early signs of cancer can conceal from suspicious eyes under the name of chronic trauma.

In the following years, such hypothesis has been repeatedly reported advocating preclinical data to support a presumptive role of chronic trauma in oral carcinogenesis. The first *in vivo* evidence of a correlation between trauma and oral carcinoma dates back to the seventies and early eighties, when animal models of oral carcinogenesis were being developed (Eveson & MacDonald, 1981; Fujita et al., 1973a,1973b; Furukawa, 1972; Marefat & Shklar, 1977). In most cases, these models used hamsters and investigated the role of mechanical trauma, as scratching with a root‐canal barbed broach of the oral mucosa before the application by painting with a sable brush of a carcinogen agent, typically 7,12‐dimethylbenzanthracene (DMBA) (Eveson & MacDonald, 1981; Fujita et al., 1973a,1973b; Furukawa, 1972; Marefat & Shklar, 1977; Omura et al., 1999). Just one paper reported a rat model and applied topically N‐nitroso‐N‐methyl‐urea (NMU) (Matiakis et al., 1994). Fujita et al. first highlighted the role of trauma in speeding up the process of tumour development, especially at the lateral border of the tongue (Fujita et al., 1973a). In all these studies, mechanical trauma was considered a co‐carcinogenic event, thus promoting the action of pro‐carcinogen agent, making the development of carcinoma faster. On the contrary, the same literature supported as the presence of trauma alone did not induce carcinoma, but histopathological findings of hyperkeratosis, with or without dysplasia (Marefat & Shklar, 1977).

Further studies, in the eighties, focused on the impact of ‘excisional wounding’ in favouring the DMBA‐related oral carcinogenesis: in this model, the middle portions (2 mm in longitudinal thickness) lingual tips were removed after preliminary topical application of DMBA. Then, the resulting wounds were further treated with DMBA. Maeda and co‐workers found that excisional wounding could act as a tumour‐promoting stimulus in association with the carcinogen agent (Maeda & Kameyama, 1986; Maeda et al., 1989). These findings were consistent with other studies on cutaneous and hepatic carcinomas, which identified abrasion and wounding as promoters in carcinogenesis using different experimental animal studies (Argyris, 1980; Argyris & Slaga, 1981; Craddock, 1975; Pound & McGuire, 1978).

A potential role for chronic trauma in carcinogenesis has been suggested, through mechanisms involving the disruption of the normal physical architecture of the extracellular matrix promoting oncogene expression, an hyperproliferative status, a favouring inflammatory micro‐environment and a facilitated exposure to carcinogens (Evans et al., 2019; Piemonte et al., 2010; Warnakulasuriya, 2009). From the seventies to the nineties, several authors addressed the association between cancer and trauma in different areas of the human body, but available data are mostly based on single case reports or come from retrospective studies, which assessed whether the onset of the tumour could be related to a previous trauma. Such observations suggested that a physically altered extracellular matrix accompanied by a loaded tissue‐specific oncogene able to elicit uncontrolled proliferation could have a role in carcinogenesis (Evans et al., 2019).

Basal cell carcinoma (BCC), one of the most encountered cutaneous malignancy, has also correlated to acute traumatism. Between 1979 and 1986, 1774 BCCs were evaluated for association with a previous trauma, such as burns, sharp and blunt trauma, but also chicken pox scars and vaccination sites. The authors observed that 129/1774 (7.3%) of the lesions had a positive association with trauma. In addition, the lesions were larger pre‐ and post‐operatively, they had required several surgical interventions indicating a clinical deceptive behaviour when compared to those of the non‐trauma group. However, the BCC lesions associated with trauma were not characterized by an aggressive histological type (Noodleman & Pollack, 1986).

Several reports about melanoma formation following a single injury of the skin or arising from thermal burn scar can be found (Akiyama et al., 1997; Alconchel et al., 1997; Delaunay et al., 1991; Gellin & Epstein, 1975; Ikeda et al., 1995; Kaskel et al., 2000; Lee et al., 2019; Muhlemann et al., 1982; Nancarrow, 1979; Stilwell & Sclare, 1980). More recently, a retrospective standardized questionnaire was administered to 369 patients affected by melanoma in order to detect a possible association between a single or recurrent traumatic event and melanoma characteristics. Thirty‐two of the 369 patients (8.7%) reported a possible correlation between the formation of the pathological lesion and trauma. In particular, 22/32 patients described a single traumatic event while the remaining 10 patients reported a persisting irritation. However, the authors concluded that there was no evidence for a causative role of trauma (single or persistent) for melanoma formation (Kaskel et al., 2000).

All these reports bring to scanty and inconclusive data. Very often the authors assessed acute rather than chronic trauma and the recall bias represents an undeniable and important limitation. When focusing on OSCC, the present review highlights the lack of strong evidence of association with chronic trauma and even more the lack of any evidence supporting an active role for chronic trauma in oral carcinogenesis, neither as promoter nor as progressor factor. From a methodological point of view, the assessment of potential association between CMI and OSCC is such an issue that it is very difficult to have level I evidence. Even lacking the recognition of CMI as a harmful oncogenic factor, ethical reasons prevent keeping in patients CMI in order to prove its role in oral carcinogenesis. The available literature comes from few retrospective case–control studies with limitations such as recall bias, selection bias, nonblinding and descriptive studies (Alburqueque et al., 2011; Balaram et al., 2002; Jham et al., 2008; Moergel et al., 2014; Overholt et al., 1996; Perry et al., 2015) lacking any controls.

Notwithstanding this, the putative association between trauma and oral cancer is mentioned by some websites, easily accessible to patients and clinicians, suggesting the role as a risk factor of intraoral traumatic events (Box 1). Some of these belong to scientific association dedicated to dental professionals or researcher working on cancer, whose mission is communication and scientific dissemination. The local trauma is cited as ‘trauma of the inner surface of the mouth, such as, for example, the incorrect positioning of dental prostheses’ or as local ‘microtrauma’ and it is placed alongside more well‐known and scientifically sound risk factors such as smoking and alcohol abuse.

BOX 1 **Websites reporting intraoral traumatic events as risk factors associated with OSCC (accessed on January 3rd, 2021)**

**Table:** 

Editor	URL
The Oral Cancer Foundation	https://oralcancerfoundation.org/understanding/risk‐factors/
AIRC (Italian Association for Cancer Research)	https://www.airc.it/cancro/informazioni‐tumori/guida‐ai‐tumori/tumore‐della‐bocca
AIDI (Italian Association of Dental Hygienists)	https://www.aiditalia.it/wp‐content/uploads/2018/02/Carcinoma‐orale‐Manuale‐di‐riferimento‐1.pdf
IERO (European Institute for Dental Research)	https://www.iero.it/il‐cancro‐alla‐bocca
Slowdentistry Network	http://www.slowdentistry.it/archives/1198
NHS (National Health Service – UK)	https://www.nhs.uk/conditions/mouth‐cancer/causes/
Medical News Today (Healthline Media UK)	https://www.medicalnewstoday.com/articles/165331
The Economic Times	https://economictimes.indiatimes.com/magazines/panache/have‐a‐sharp‐tooth‐dont‐skip‐your‐dental‐appointment‐its‐linked‐to‐oral‐cancer/articleshow/65018684.cms?from=mdr
Ontario Dental Association	https://www.youroralhealth.ca/patient‐education‐materials/147‐oral‐health‐your‐body/oral‐cancer
FDI World Dental Federation	https://www.fdiworlddental.org/sites/default/files/media/resources/fdi‐oral_cancer‐prevention_and_patient_management.pdf
HMP ‐ Population Health Learning Network	https://www.managedhealthcareconnect.com/content/rough‐teeth‐and‐rubbing‐dentures‐may‐be‐linked‐oral‐cancer

Websites reporting intraoral traumatic events as risk factors associated with OSCC (accessed on January 03rd 2021).

On the contrary, just the website of the Oral Cancer Foundation goes in the opposite direction, explicitly excluding this kind of association and highlighting the importance of ‘reassuring patients that dental appliances, restorative materials, and routine trauma do not appear to increase the probability of oral cancer’ (Box 1).

Chronic mucosal trauma is suggested as an additional etiologic risk factor for OSCC, but there is a lack of both clinical and experimental‐molecular data available to support such hypothesis and studies are not free from several shortcomings. Loss of heterozygosity (LOH) is an early genetic alteration suggesting potential progression towards OSCC; a recent study investigating the presence of LOH (arms 3p, 9p and 17p) in inflammatory fibrous hyperplasia associated with removable dental prosthesis, did not find any evidence to support the role of trauma in initiating carcinogenesis via persistent DNA damage that might lead to the accumulation of genetic mutations (Bernardes et al., 2019). Nevertheless, several flaws should be cited, among which the authors did not specified whether the lesions were active, or residual, and the low number of cases prevented any statistical analysis.

The presence of one single paper fulfilling the inclusion criteria represents the main limitation of the present review. Therefore, the available evidence is limited, and it does not appear to be significant enough to support broken/sharp teeth or wearing denture to be risk factors in causing oral cancer.

Other narrative (Singhvi et al., 2017) or systematic reviews (Gupta et al., 2021; Manoharan et al., 2014) investigated this association, but none reported the adopted criteria to define exposure to CMI. This conceivably led to the inclusion of studies lacking any restrictive definition of trauma and therefore to an overestimation of the association between CMI and OSCC. The meta‐analysis by Manoharan S et al reported that ill‐fitting dentures are a risk factor for the development of oral cancer. Nevertheless, the analysis included any studies reporting a generic use of denture and it did not clarify in which cases dentures were considered ill‐fitting, neither clarified how nor from which of the selected studies data on ill‐fitting denture were extracted (Manoharan et al., 2014). Gupta AA et al included case–control and cohort studies investigating CMI irrespective of any specific definition of trauma. Even using non‐restrictive criteria, the authors were able to identify a significant association between CMI and OSCC, but they were not able to assess CMI as an independent risk factor for OSCC (Gupta et al., 2021). Our approach in selecting the studies to be included in the review, certainly more restrictive and rigorous, highlights that there is still no strong, high‐quality evidence to support a causative role of chronic mucosal trauma in oral carcinogenesis. Similarly, there is no evidence to consider chronic ulcer or chronic mucosal irritation, as part of OPMDs as suggested by some authors (Lazos et al., 2019; Panta et al., 2018). Of course, the detection of chronic trauma should lead to its removal as a good clinical practice, but labelling conditions of dental or denture related trauma as OPMDs would leave pass to misconceptions in pathogenesis and potentially dangerous medico‐legal issues.

Looking for evidence based on sound definition of CMI, the literature does neither support nor exclude an active role for chronic trauma in oral carcinogenesis, neither as promoter nor as progressor factor. Notwithstanding this, clinical conditions associated with CMI (e.g. frictional keratosis) are currently recognized as non‐part of the oral potentially malignant disorders. Prospective cohort studies able to better assess trauma in OSCC are needed.

## CONFLICTS OF INTEREST

None to declare.

## ACKNOWLEDGEMENT

Open Access Funding provided by Universita degli Studi dell'Insubria within the CRUI‐CARE Agreement.

## AUTHOR CONTRIBUTIONs

**Monica Pentenero:** Conceptualization; Data curation; Investigation; Project administration; Software; Writing‐original draft. **Lorenzo Azzi:** Data curation; Formal analysis; Investigation; Writing‐original draft. **Giovanni Lodi:** Data curation; Investigation; Methodology; Validation; Writing‐original draft. **Maddalena Manfredi:** Data curation; Formal analysis; Investigation; Writing‐original draft. **Elena Maria Maria Varoni:** Data curation; Investigation; Supervision; Writing‐original draft; Writing‐review & editing.

### PEER REVIEW

The peer review history for this article is available at https://publons.com/publon/10.1111/odi.14049.

## Supporting information

Supplementary Material
