# Supplementary material for: Chronic mechanical trauma/irritation and oral carcinoma: A systematic review showing low evidence to support an association
Source: Oral Dis. 2021 Oct 20;28(8):2110–8. doi: 10.1111/odi.14049 (PMC9787889; doi:10.1111/odi.14049)
Supplement: Supplementary file 1 — Supplementary Material [file ODI-28-2110-s001.pdf]

Queries

<u>PubMed</u>

((("cohort studies"[MeSH Terms:noexp] OR (cohort[Title/Abstract] AND study[Title/Abstract]) OR (cohort[Title/Abstract] AND studies[Title/Abstract])) OR ("case-control studies"[MeSH Terms] OR ("case-control"[All Fields] AND "studies"[All Fields]) OR "case-control studies"[All Fields] OR ("case"[All Fields] AND "control"[All Fields]) OR "case control"[All Fields])) OR ("cross-sectional studies"[MeSH Terms] OR ("cross-sectional"[All Fields] AND "studies"[All Fields]) OR "cross-sectional studies"[All Fields] OR ("cross"[All Fields] AND "sectional"[All Fields] AND "study"[All Fields]) OR "cross sectional study"[All Fields])) AND ("mouth neoplasms"[MeSH Terms] OR ("mouth"[All Fields] AND "neoplasms"[All Fields]) OR "mouth neoplasms"[All Fields] OR "oral cancer"[All Fields])

<u>EMBASE</u>

('mouth tumor'/exp OR 'mouth tumor' OR 'mouth cancer'/exp OR 'mouth cancer') AND ('cohort analysis' OR 'case control study' OR 'cross-sectional study')

Characteristics of Included studies

(Piemonte et al, 2018)

- Study design: prospective case-control
- Country: Argentina
- Time of recruitment: 2009-2013
- Setting: University Dental Clinic
- Number of participants: 153
  OSCC group: 53; Control group: 100
- Inclusion criteria for OSCC group: squamous cell, in situ and verrucous carcinoma, confirmed histopathologically; for control group: subjects seeking dental treatment at the same institution during the same period

<u>*Risk of bias assessment*</u>:

The Joanna Briggs Institute Critical Appraisal Checklist for case-control studies includes 10 questions exploring different domains of the case-control study design, focusing on the group selection and matching, the methods of measurement of exposure, the assessment of the confounding factors, and the statistical analysis.

	Yes	No	Unclear	Not applicable
1. Were the groups comparable other than the presence of disease in cases or the absence of disease in controls?		X		
2. Were cases and controls matched appropriately?		X		
3. Were the same criteria used for identification of cases and controls?			X	
4. Was exposure measured in a standard, valid and reliable way?		X		

5. Was exposure measured in the same way for cases and controls?		X
6. Were confounding factors identified?	X	
7. Were strategies to deal with confounding factors stated?	X	
8. Were outcomes assessed in a standard, valid and reliable way for cases and controls?		X
9. Was the exposure period of interest long enough to be meaningful?		X
10. Was appropriate statistical analysis used?	X	

The number of both OSCC and control subjects exposed to CMI is not consistently reported in the tables of the paper. In table 3 exposed OSCC subject are 38 out of 53 and exposed controls are 36 out of 100, leading to an OR of 4.5 (95% CI 2.2-9.2). Conversely, in table 1 exposed OSCC subject are 31 and exposed controls are 37.

Overall assessment: HIGH RISK

Characteristics of Excluded studies

(Alburqueque et al, 2011)
This cross-sectional study lacked a control group recruiting healthy subjects.

(Balaram et al, 2002)
Absence of histological diagnosis; absence of a control group; no clinical data on OSCC available

(Bektas-Kayhan et al, 2014)
Absence of clinical information on topographical relationship between cancer and dental trauma

(Brouha et al, 2005)
Chronic mechanical irritation was not investigated, and the study lacked a control group

(Campbell et al, 1997)
The study group was composed only by patients affected by alveolar ridge carcinoma, whereas the control group was composed by subjects affected by other kinds of oral or head and neck cancers.

(Garrote et al, 2001)
No data about trauma, only duration of denture wearing

(Gellrich et al, 2003)
This study showed only a survey among oncological patients and clinicians

(Gillison, 2007)
This was a review paper

(Guha et al, 2007)
Absence of clinical data on mechanical irritation

(Guneri et al, 2005)
Data were collected by questionnaires and not assessed through a clinical examination.

(Jain et al, 2016)
Data were collected by questionnaires and not assesses through a clinical examination.

(Jham et al, 2008)
Retrospective descriptive study without control group

(Lazos et al, 2017)
Cross-sectional study with selected groups for comparison (chronic traumatic ulcer, benign irritative mechanical lesions)

(Marshall et al, 1992)
No data about trauma, only duration of denture wearing

(Manoharan et al, 2014)
Systematic review and meta-analysis

(Moergel et al, 2014)
Incidence of OSCC near to dental implants, off-topic. Absence of a control group

(Overholt et al, 1996)
Retrospective descriptive study, absence of control group

(Perry et al, 2015)
Retrospective descriptive study, absence of control group

(Piemonte et al, 2010)
Absence of patients with chronic traumatic ulcers from the control group

(Rosenquist et al, 2005)
Off-topic, absence of clinical data on chronic mechanical trauma

(Rotundo et al, 2013)
Absence of clinical data, administration of a questionnaire to recruited subjects

(Talamini et al, 2000)
No data about trauma, only duration of denture wearing

(Tezal et al, 2005)
Off-topic; survey on periodontal features related to oral cancer

(Vaccarezza et al, 2010)
Self-reporting, no clinical data

(Velly et al, 1998)

Case-control study based on interviews

(Winn et al, 1991)
Case-control study based on interviews

(Yan et al, 2017)
Absence of clinical data, interview

(Zheng et al, 1990)
Clinical data on OSCC and topographical relationship not available

(Zheng et al, 1992)
Clinical data on OSCC and topographical relationship not available

(Zuo et al, 2015)
This study focused on the loss of teeth, but not on chronic mechanical irritation; off-topic

Characteristics of Excluded studies (specifically reassessed because reported in previous reviews from the literature)

(Thumfart *et al*, 1978)
The study lacked a control group

(Gorsky and Silverman, 1984)
The study lacked a control group

(Young *et al*, 1986)
Control group consisted of patients affected by cancer of larynx

(Franco *et al*, 1989)
Absence of clinical data, administration of a questionnaire to recruited subjects

(Lockhart *et al*, 1998)
The control group consisted of patients affected by extra-oral malignancies

(Schildt *et al*, 1998)
Absence of clinical data, administration of a questionnaire to recruited subjects

(Fan *et al*, 2015)
The study lacked a control group

(Bernardes *et al*, 2019)
Histological study

References

Alburqueque R, Lopez-Lopez J, Mari-Roig A, Jane-Salas E, Chimenos-Kustner E and Santos JR (2011). Relationship between squamous cell carcinoma of the anterior two thirds of the tongue and removable denture use: a pioneer study in a Portuguese population. *Brazilian dental journal* **22**: 410-4.

Balaram P, Sridhar H, Rajkumar T, Vaccarella S, Herrero R, Nandakumar A, Ravichandran K, Ramdas K, Sankaranarayanan R, Gajalakshmi V, Munoz N and Franceschi S (2002). Oral cancer in southern India: the influence of smoking, drinking, paan-chewing and oral hygiene. *International journal of cancer* **98**: 440-5.

Bektas-Kayhan K, Karagoz G, Kesimli MC, Karadeniz AN, Meral R, Altun M and Unur M (2014). Carcinoma of the tongue: a case-control study on etiologic factors and dental trauma. *Asian Pac J Cancer Prev* **15**: 2225-9.

Bernardes VF, Diniz MG, Silva JC, Moraes DC, De Marco L, Gomes CC and Gomez RS (2019). Lack of association between denture trauma and loss of heterozygosity confronts the proposed pathologic role of chronic mucosal trauma in oral carcinogenesis. *J Oral Pathol Med* **48**: 421-423.

Brouha XD, Tromp DM, Hordijk GJ, Winnubst JA and de Leeuw JR (2005). Oral and pharyngeal cancer: analysis of patient delay at different tumor stages. *Head & neck* **27**: 939-45.

Campbell BH, Mark DH, Soneson EA, Freije JE and Schultz CJ (1997). The role of dental prostheses in alveolar ridge squamous carcinomas. *Archives of otolaryngology--head & neck surgery* **123**: 1112-5.

Fan H, Yoon KY, Kim SM, Myoung H, Lee JH and Kim MJ (2015). Relationship between squamous cell carcinoma of the tongue and the position of dental prosthesis. *J Adv Prosthodont* **7**: 129-37.

Franco EL, Kowalski LP, Oliveira BV, Curado MP, Pereira RN, Silva ME, Fava AS and Torloni H (1989). Risk factors for oral cancer in Brazil: a case-control study. *International journal of cancer* **43**: 992-1000.

Garrote LF, Herrero R, Reyes RM, Vaccarella S, Anta JL, Ferbeye L, Munoz N and Franceschi S (2001). Risk factors for cancer of the oral cavity and oro-pharynx in Cuba. *Br J Cancer* **85**: 46-54.

Gellrich NC, Suarez-Cunqueiro MM, Bremerich A and Schramm A (2003). Characteristics of oral cancer in a central European population: defining the dentist's role. *Journal of the American Dental Association (1939)* **134**: 307-14; quiz 338.

Gillison ML (2007). Current topics in the epidemiology of oral cavity and oropharyngeal cancers. *Head & neck* **29**: 779-92.

Gorsky M and Silverman S, Jr. (1984). Denture wearing and oral cancer. *J Prosthet Dent* **52**: 164-6.

Guha N, Boffetta P, Wunsch Filho V, Eluf Neto J, Shangina O, Zaridze D, Curado MP, Koifman S, Matos E, Menezes A, Szeszenia-Dabrowska N, Fernandez L, Mates D, Daudt AW, Lissowska J, Dikshit R and Brennan P (2007). Oral health and risk of squamous cell carcinoma of the head and neck and esophagus: results of two multicentric case-control studies. *American journal of epidemiology* **166**: 1159-73.

Guneri P, Cankaya H, Yavuzer A, Guneri EA, Erisen L, Ozkul D, El SN, Karakaya S, Arican A and Boyacioglu H (2005). Primary oral cancer in a Turkish population sample: association with sociodemographic features, smoking, alcohol, diet and dentition. *Oral Oncol* **41**: 1005-12.

Jain P, Jain M, Prasad BV, Kakatkar GS, Patel M and Khan J (2016). A Case-control Study for the Assessment of Correlation of Denture-related Sores and Oral Cancer Risk. *The journal of contemporary dental practice* **17**: 930-933.

Jham BC, Reis PM, Miranda EL, Lopes RC, Carvalho AL, Scheper MA and Freire AR (2008). Oral health status of 207 head and neck cancer patients before, during and after radiotherapy. *Clinical oral investigations* **12**: 19-24.

Lazos JP, Piemonte ED, Lanfranchi HE and Brunotto MN (2017). Characterization of Chronic Mechanical Irritation in Oral Cancer. *Int J Dent* **2017**: 6784526.

Lockhart PB, Norris CM, Jr. and Pulliam C (1998). Dental factors in the genesis of squamous cell carcinoma of the oral cavity. *Oral Oncol* **34**: 133-9.
Manoharan S, Nagaraja V and Eslick GD (2014). Ill-fitting dentures and oral cancer: a meta-analysis. *Oral Oncol* **50**: 1058-61.
Marshall JR, Graham S, Haughey BP, Shedd D, O'Shea R, Brasure J, Wilkinson GS and West D (1992). Smoking, alcohol, dentition and diet in the epidemiology of oral cancer. *European journal of cancer. Part B, Oral oncology* **28b**: 9-15.
Moergel M, Karbach J, Kunkel M and Wagner W (2014). Oral squamous cell carcinoma in the vicinity of dental implants. *Clinical oral investigations* **18**: 277-84.
Overholt SM, Eicher SA, Wolf P and Weber RS (1996). Prognostic factors affecting outcome in lower gingival carcinoma. *The Laryngoscope* **106**: 1335-9.
Perry BJ, Zammit AP, Lewandowski AW, Bashford JJ, Dragovic AS, Perry EJ, Hayatbakhsh R and Perry CF (2015). Sites of origin of oral cavity cancer in nonsmokers vs smokers: possible evidence of dental trauma carcinogenesis and its importance compared with human papillomavirus. *JAMA otolaryngology-- head & neck surgery* **141**: 5-11.
Piemonte E, Lazos J, Belardinelli P, Secchi D, Brunotto M and Lanfranchi-Tizeira H (2018). Oral cancer associated with chronic mechanical irritation of the oral mucosa. *Med Oral Patol Oral Cir Bucal* **23**: e151-e160.
Piemonte ED, Lazos JP and Brunotto M (2010). Relationship between chronic trauma of the oral mucosa, oral potentially malignant disorders and oral cancer. *J Oral Pathol Med* **39**: 513-7.
Rosenquist K, Wennerberg J, Schildt EB, Bladstrom A, Goran Hansson B and Andersson G (2005). Oral status, oral infections and some lifestyle factors as risk factors for oral and oropharyngeal squamous cell carcinoma. A population-based case-control study in southern Sweden. *Acta Otolaryngol* **125**: 1327-36.
Rotundo LD, Toporcov TN, Biazevic GH, de Carvalho MB, Kowalski LP and Antunes JL (2013). Are recurrent denture-related sores associated with the risk of oral cancer? A case control study. *Revista brasileira de epidemiologia = Brazilian journal of epidemiology* **16**: 705-15.
Schildt EB, Eriksson M, Hardell L and Magnuson A (1998). Oral infections and dental factors in relation to oral cancer: a Swedish case--control study. *Eur J Cancer Prev* **7**: 201-6.
Talamini R, Vaccarella S, Barbone F, Tavani A, La Vecchia C, Herrero R, Munoz N and Franceschi S (2000). Oral hygiene, dentition, sexual habits and risk of oral cancer. *Br J Cancer* **83**: 1238-42.
Tezal M, Grossi SG and Genco RJ (2005). Is periodontitis associated with oral neoplasms? *Journal of periodontology* **76**: 406-10.
Thumfart W, Weidenbecher M, Waller G and Pesch HG (1978). Chronic mechanical trauma in the aetiology of oro-pharyngeal carcinoma. *J Maxillofac Surg* **6**: 217-21.
Vaccarezza GF, Antunes JL and Michaluart-Junior P (2010). Recurrent sores by ill-fitting dentures and intra-oral squamous cell carcinoma in smokers. *Journal of public health dentistry* **70**: 52-7.
Velly AM, Franco EL, Schlecht N, Pintos J, Kowalski LP, Oliveira BV and Curado MP (1998). Relationship between dental factors and risk of upper aerodigestive tract cancer. *Oral Oncol* **34**: 284-91.
Winn DM, Blot WJ, McLaughlin JK, Austin DF, Greenberg RS, Preston-Martin S, Schoenberg JB and Fraumeni JF, Jr. (1991). Mouthwash use and oral conditions in the risk of oral and pharyngeal cancer. *Cancer Res* **51**: 3044-7.
Yan L, Chen F, He B, Liu F, Liu F, Huang J, Wu J, Lin L, Qiu Y and Cai L (2017). A novel environmental exposure index and its interaction with familial susceptibility on oral cancer in non-smokers and non-drinkers: a case-control study. *Eur Arch Otorhinolaryngol* **274**: 1945-1950.
Young TB, Ford CN and Brandenburg JH (1986). An epidemiologic study of oral cancer in a statewide network. *Am J Otolaryngol* **7**: 200-8.

Zheng TZ, Boyle P, Hu HF, Duan J, Jian PJ, Ma DQ, Shui LP, Niu SR, Scully C and MacMahon B (1990). Dentition, oral hygiene, and risk of oral cancer: a case-control study in Beijing, People's Republic of China. *Cancer Causes Control* **1**: 235-41.

Zheng W, Blot WJ, Shu XO, Diamond EL, Gao YT, Ji BT and Fraumeni JF, Jr. (1992). Risk factors for oral and pharyngeal cancer in Shanghai, with emphasis on diet. *Cancer epidemiology, biomarkers & prevention : a publication of the American Association for Cancer Research, cosponsored by the American Society of Preventive Oncology* **1**: 441-8.

Zuo C, Zhu Y, Wang X, Zeng X and Huang C (2015). Tooth loss and risk of oral squamous cell carcinoma in Chinese Han population. *International journal of clinical and experimental medicine* **8**: 21893-7.
